# Supplementary material for: Translation of small downstream ORFs enhances translation of canonical main open reading frames
Source: EMBO J. 2020 Aug 3;39(17):e104763. doi: 10.15252/embj.2020104763 (PMC7459409; doi:10.15252/embj.2020104763)
Supplement: Supplementary file 1 — Expanded View Figures PDF [file EMBJ-39-e104763-s001.pdf]

Expanded View Figures

Figure EV1. Identification of translated dORFs using ribosome profiling.

A Cartoon for defining potential dORF in 3′ UTR based on transcriptome sequence. We searched for start–stop codon pairs within 10–100 amino acids in all 3 reading frames defined based on canonical ORF. For the start codon, the most distal ATG was first considered as a possible start codon, followed by non-ATG (CTG, GTG, TTG) start codons in non-overlapping regions, dark blue indicates ATG, light blue indicates non-ATG, and gray indicates stop codon.

B Scatter plot for ORFscore and in-frame proportion ribosome footprint coverage for all ORFs: annotated CDS (green), 5′ UTR uORFs (purple), 3′ UTR dORFs (red), and ORFs overlapping the annotated CDS (orange).

C Pie chart for distribution of all human translated dORFs based on start codon of the dORF.

D Metagene plots showing the distribution of the ribosome footprint and input RNA reads around the start and stop codons of canonical ORF and dORF in zebrafish high-confidence dORF-containing genes. The ribosome footprint reads mainly show the characteristic 3-nucleotide periodicity across the translated ORF, while the RNA shows uniform distribution across the mRNA. Green indicates the canonical ORF; blue indicates the internal UTR (iUTR) between the stop codon of the canonical ORF and the start codon of the dORF; and red indicates the dORF. Insite shows ribosome distribution at the dORF region, close to its start and stop codon.

E Metagene plots of ribosome footprint reads around the start and stop codon of dORFs in human and zebrafish genes, for ATG dORFs with different confidence levels (high, middle, and low). Blue indicates iUTR; red indicates dORF.

F Metagene plots of ribosome footprint reads around the start and stop codon of dORFs in human and zebrafish genes, for non-ATG dORFs with different confidence levels (high, middle, and low). Blue indicates iUTR; red indicates dORF.

 © 2020 The Authors

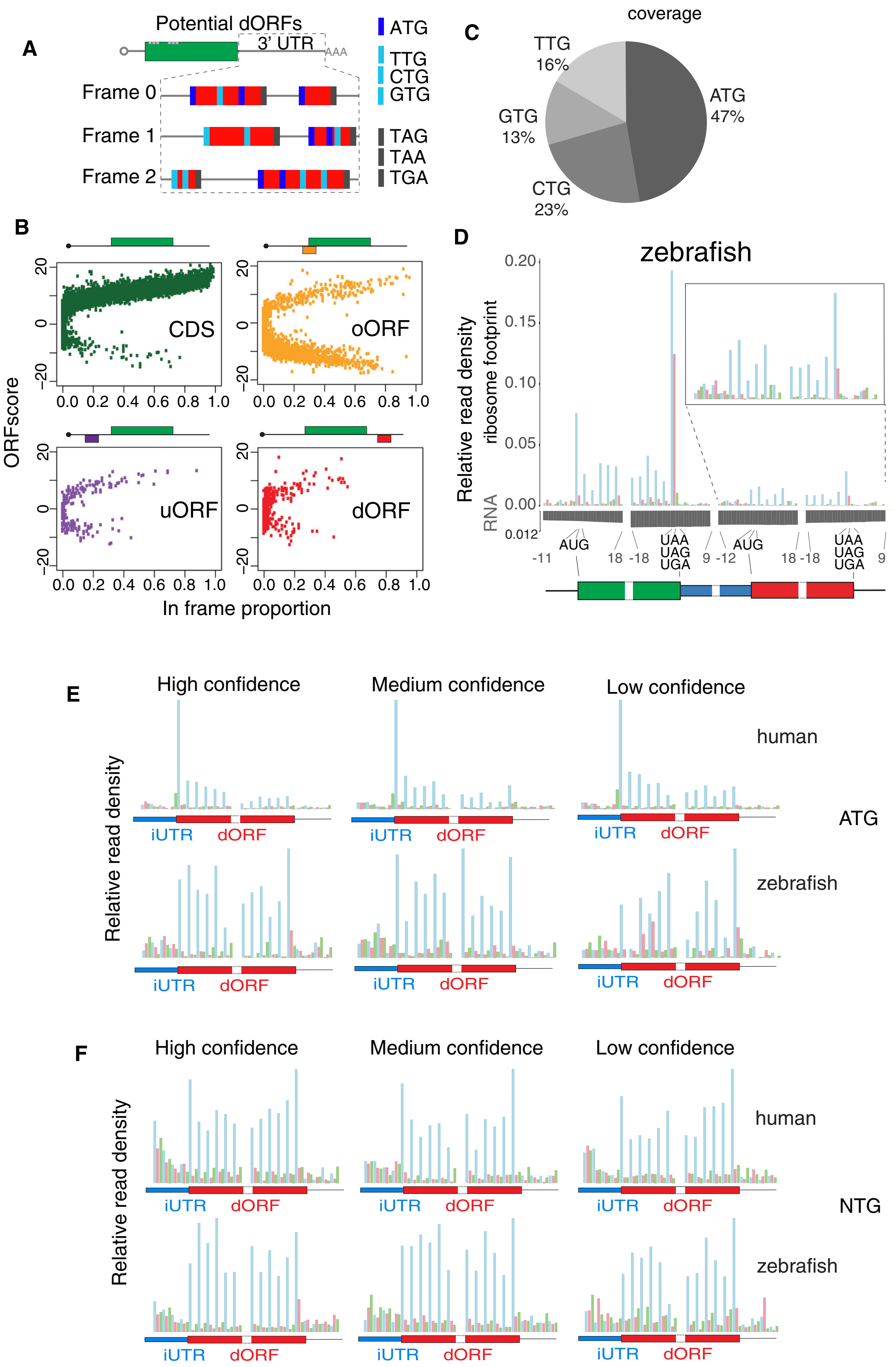
A
Potential dORFs
3' UTR
AAA
Frame 0
Frame 1
Frame 2
ATG
TTG
CTG
GTG
TAG
TAA
TGA
B
ORFscore
CDS
oORF
uORF
dORF
In frame proportion
C
coverage
TTG 16%
GTG 13%
CTG 23%
ATG 47%
D
zebrafish
Relative read density
ribosome footprint
RNA
AUG
UAA UAG UGA
iUTR
dORF
E
High confidence
Medium confidence
Low confidence
human
zebrafish
ATG
F
NTG

Figure EV1.

Figure EV2. All groups of mRNAs, based on the dORF identification confidence level and/or translation start codon, display higher translation efficiency.

A Boxplot for length of dORF and iUTR for ATG and non-ATG dORF with translation evidences as well as random (light gray) and all dORF (dark gray) with no evidences of translation in zebrafish.

B Boxplot for RNA coverage differences in human and zebrafish high-confidence dORFs between each of the indicating regions of the mRNA and the last 100 nt of canonical ORF for each gene. The median of both group of mRNAs, containing translated dORF (red) or none translated dORF (gray), is close to 0, indicating uniform RNA read distribution cross the RNA, and supporting the idea that dORFs are not encoded by alternative isoforms.

C Histogram distribution of bootstrapped orthologous genes. Genes drawn to the same size pool as dORFs with ortholog in another species from each group (862 human and 610 zebrafish). Blue lines indicate 95% confidence interval (64–98); red line indicates actual orthologous genes (123).

D Cumulative distribution of mRNA level and translation efficiency of genes in zebrafish. All genes are indicated in black, and controls for mRNA containing uORF (purple) or dORF (red) were resampled to share similar mRNA level (light purple for uORF controls and orange for dORF controls). *P*-value indicated, Wilcoxon rank-sum test. Only high-confidence ATG dORFs were used in this analysis.

E Cumulative plot for RNA level and translation efficiency of genes containing ATG dORFs in human, grouped by confidence level of dORF translation (high, middle, and low), controls are resampled for similar mRNA level to compare the translation efficiency for each group. *P*-value indicated, Wilcoxon rank-sum test. Red indicates dORF gene, and gray indicates control gene.

F Cumulative plot for RNA level and translation efficiency of genes containing non-ATG dORFs in human, grouped by confidence level of dORF translation (high, middle, and low), controls are resampled for similar mRNA level to compare the translation efficiency for each group. *P*-value indicated, Wilcoxon rank-sum test. Red indicates dORF gene, and gray indicates control gene.

© 2020 The Authors

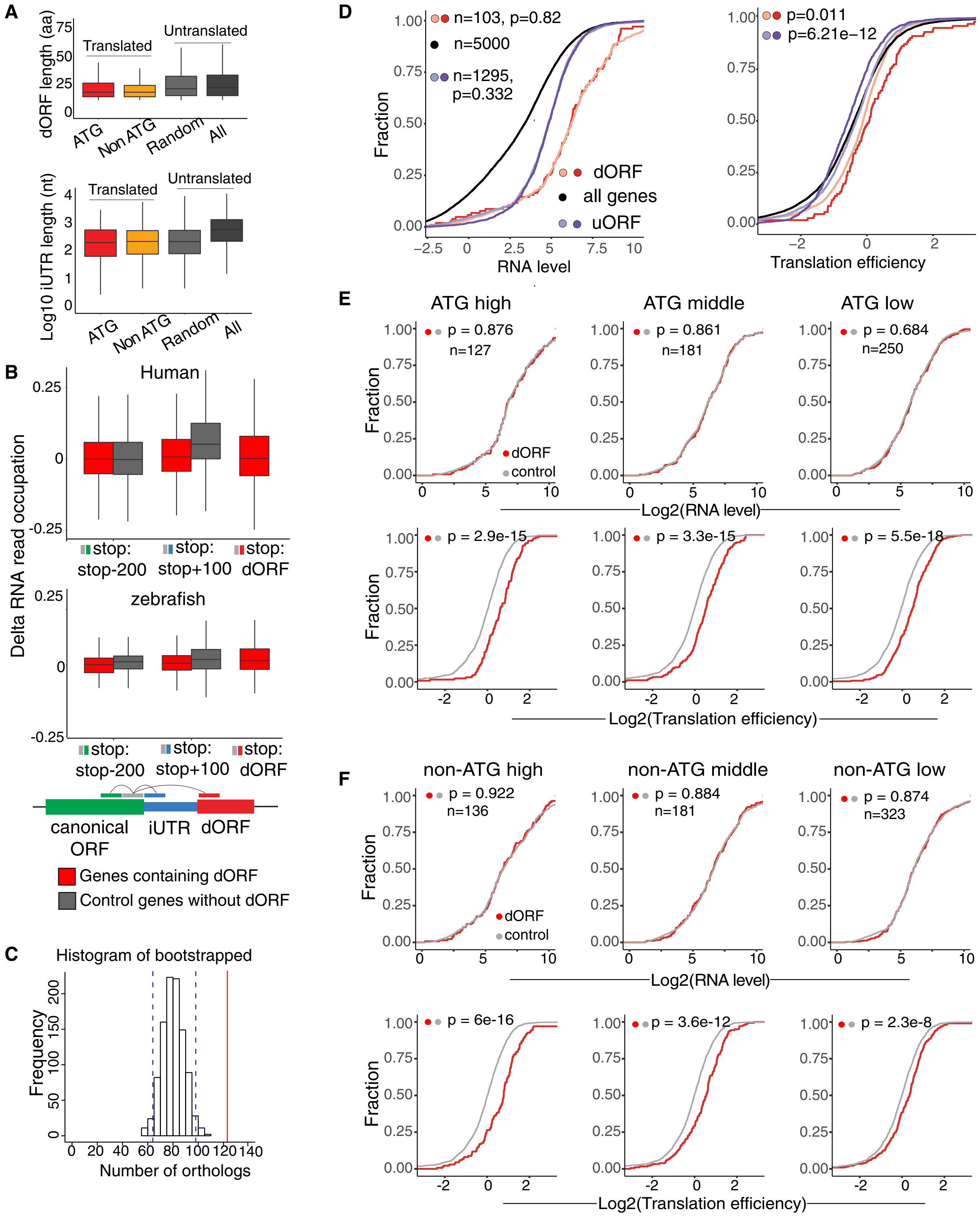
A
dORF length (aa)
Translated
Untranslated
ATG
Non ATG
Random
All
Log10 iUTR length (nt)
B
Human
Delta RNA read occupation
stop: stop-200
stop: stop+100
stop: dORF
zebrafish
canonical ORF
iUTR
dORF
Genes containing dORF
Control genes without dORF
C
Histogram of bootstrapped
Frequency
Number of orthologs
D
n=103, p=0.82
n=5000
n=1295, p=0.332
Fraction
RNA level
dORF
all genes
uORF
p=0.011
p=6.21e−12
Translation efficiency
E
ATG high
ATG middle
ATG low
p = 0.876
n=127
p = 0.861
n=181
p = 0.684
n=250
dORF
control
Log2(RNA level)
p = 2.9e-15
p = 3.3e-15
p = 5.5e-18
Log2(Translation efficiency)
F
non-ATG high
non-ATG middle
non-ATG low
p = 0.922
n=136
p = 0.884
n=181
p = 0.874
n=323
p = 6e-16
p = 3.6e-12
p = 2.3e-8

Figure EV2.

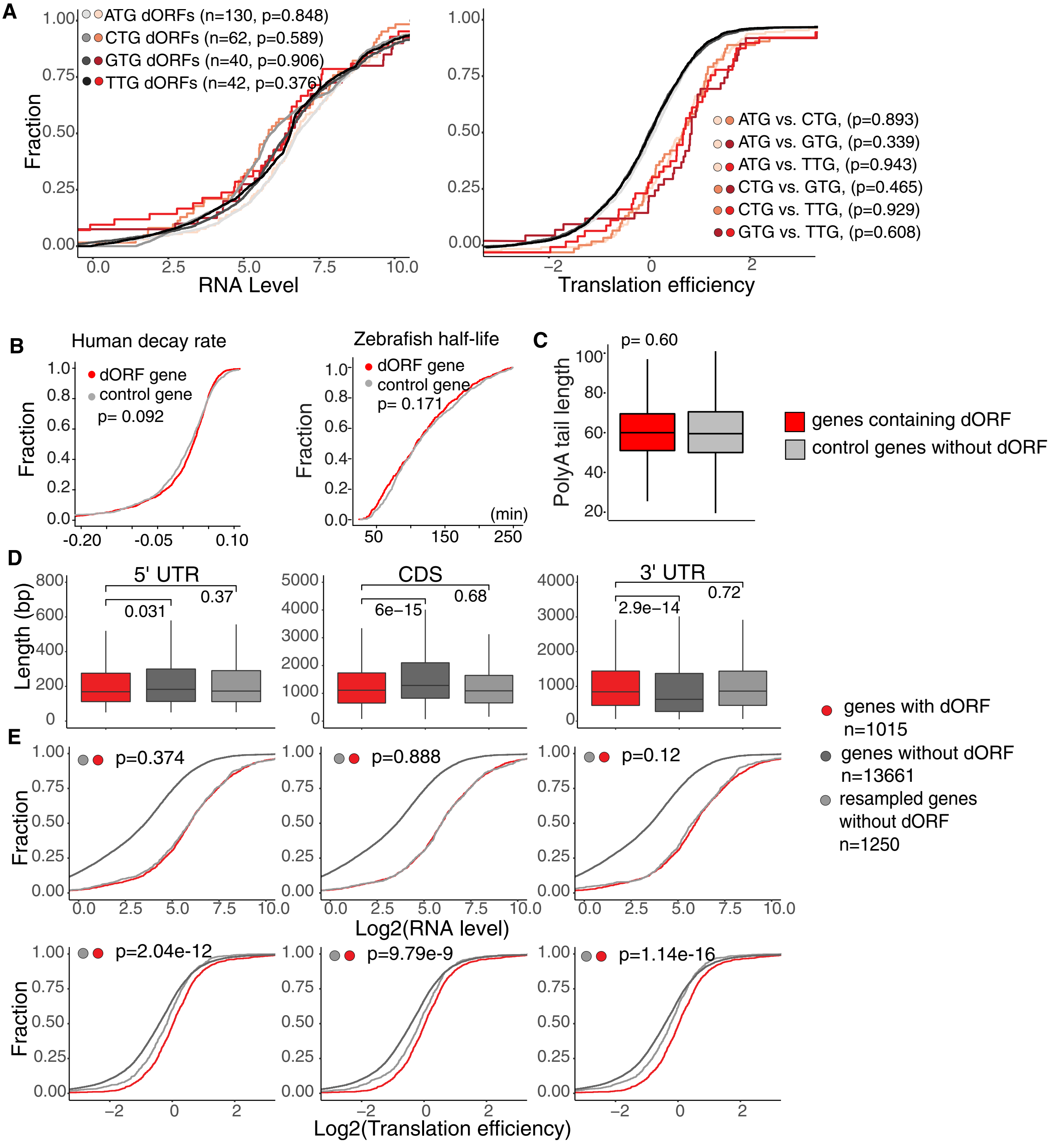

Figure EV3.

 © 2020 The Authors

Figure EV3. mRNAs with translated dORFs are efficiently translated independently of the 5′ UTR, CDS or 3′ UTR length.

A Cumulative distribution of mRNA level and translation efficiency of mRNA with human high-confidence dORFs with different translation start codons. Controls for each group were resampled to share similar mRNA level. *P*-value indicated, Wilcoxon rank-sum test. Light orange is mRNAs with ATG dORF, orange is mRNAs with CTG dORF, dark red is mRNAs with GTG dORF, and red is mRNAs with TTG dORF.

B Cumulative plot for RNA decay rate or half-life of genes containing translated dORFs and resampled controls with similar RNA level in HeLa cells and zebrafish embryos, *P*-value indicated, Wilcoxon rank-sum test. Red indicates dORF gene, and gray indicates control gene without translated dORF.

C Boxplot of median poly(A) tail length for human genes with translated dORF and resampled controls with similar RNA level in HeLa cells, *P*-value indicated, Wilcoxon rank-sum test. Red indicates dORF gene, and gray indicates control gene.

D Boxplot showing the length of 5′ UTR, CDS (canonical ORF), and 3′ UTR for zebrafish genes with translated dORF (red), all genes without translated dORF (dark gray) and resample controls without translated dORF for similar length and RNA level (light gray). *P*-value indicated, Wilcoxon rank-sum test.

E Cumulative plot for RNA level and translation efficiency of genes containing translated dORFs in zebrafish, controls are genes with no translated dORF, which are resampled for similar mRNA level, as well as similar length of either 5′ UTR (left), CDS (middle), or 3′ UTR (right) to compare translation efficiency. *P*-value indicated, Wilcoxon rank-sum test.

© 2020 The Authors

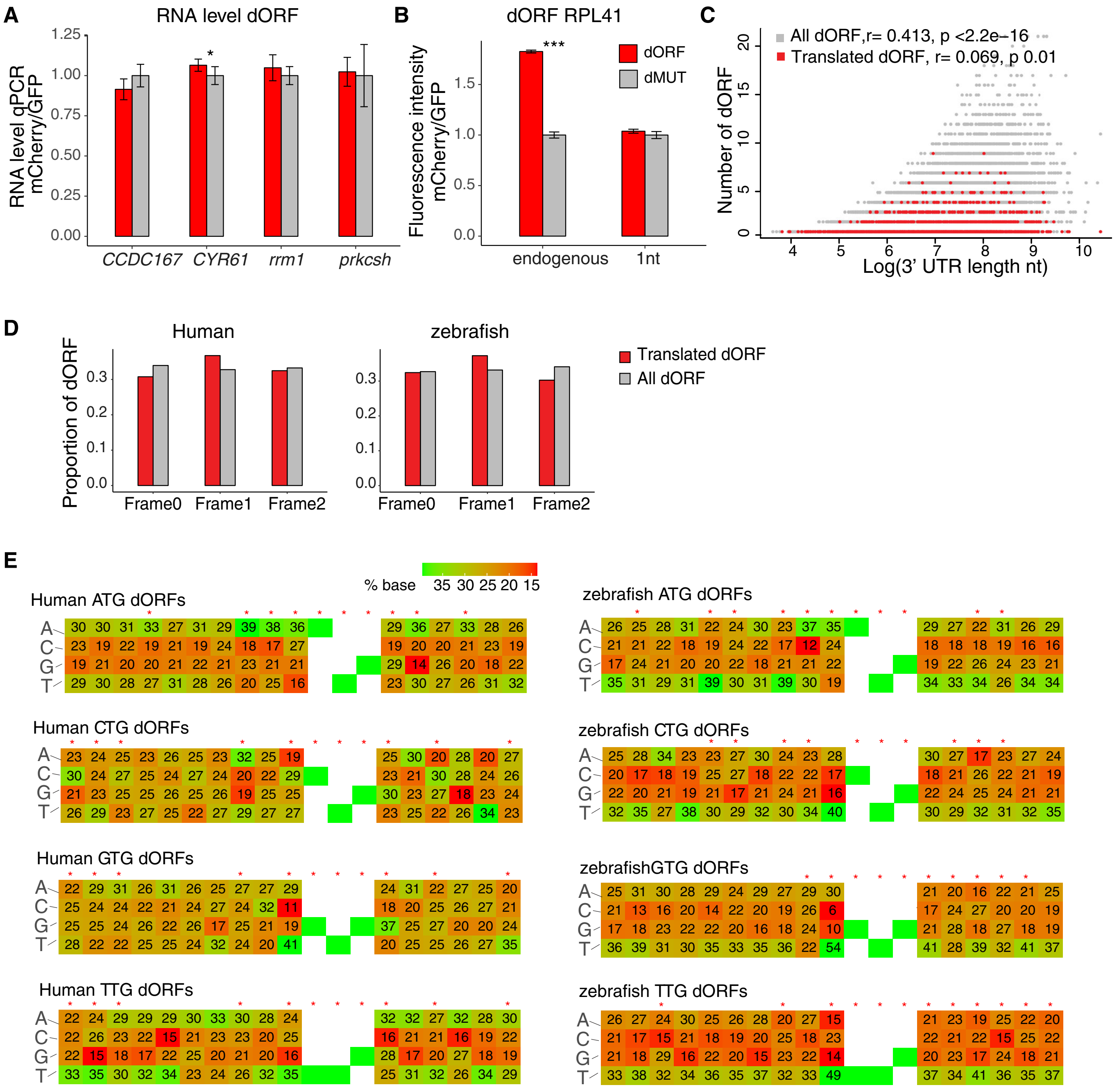

Figure EV4.

 © 2020 The Authors

Figure EV4. Characterization of dORF and iUTR sequences.

A Bar plot showing that the dORF and dMUT reporters have similar RNA level when endogenous iUTR and artificial dORF1 and paired mutation (dMUT1) as shown in Fig 4B were transfected with DNA.

B Bar plot of reporters with *RPL41* gene endogenous iUTR and endogenous dORF or artificial dORF (dORF1 as shown in Fig 4B). Reporter containing endogenous dORF shows higher fluorescence intensity than its counterpart (dMUT), while reporter with artificial dORF has similar fluorescence intensity with its counterpart.

C Scatter plot showing the length of the 3′ UTR and number of dORFs in human, translated dORF is indicated in red, and all possible dORFs (translated and untranslated) are indicated in gray. *r* and *P*-value indicated, Pearson correlations were calculated.

D Bar plot for dORF frame distributions in human and zebrafish. The frames are defined by the canonical ORF, translated dORF is indicated in red, and all possible dORFs are indicated in gray.

E Sequence nearby the dORF start codon presented a significant bias compared to the nucleotide composition present in the 3′ UTRs. The number shows ratio of each nucleotide in different positions. The four translation start sites (NTG) in human and zebrafish were separately analyzed. The red asterisks indicated with position with significant nucleotide bias ($P < 0.05$, chi-squared test). ATG analysis panel is taken from Fig 6E (duplication of image).

Data information: For Fig EV4, unpaired *t*-test is used *$P < 0.05$, ***$P < 0.005$. For transfection followed by cytometry quantification, two biological replicates with two technical replicates were done; the error bar shows SD. For transfection followed by qPCR analysis, two biological replicates with three technical replicates were done; the error bar shows SD.

© 2020 The Authors

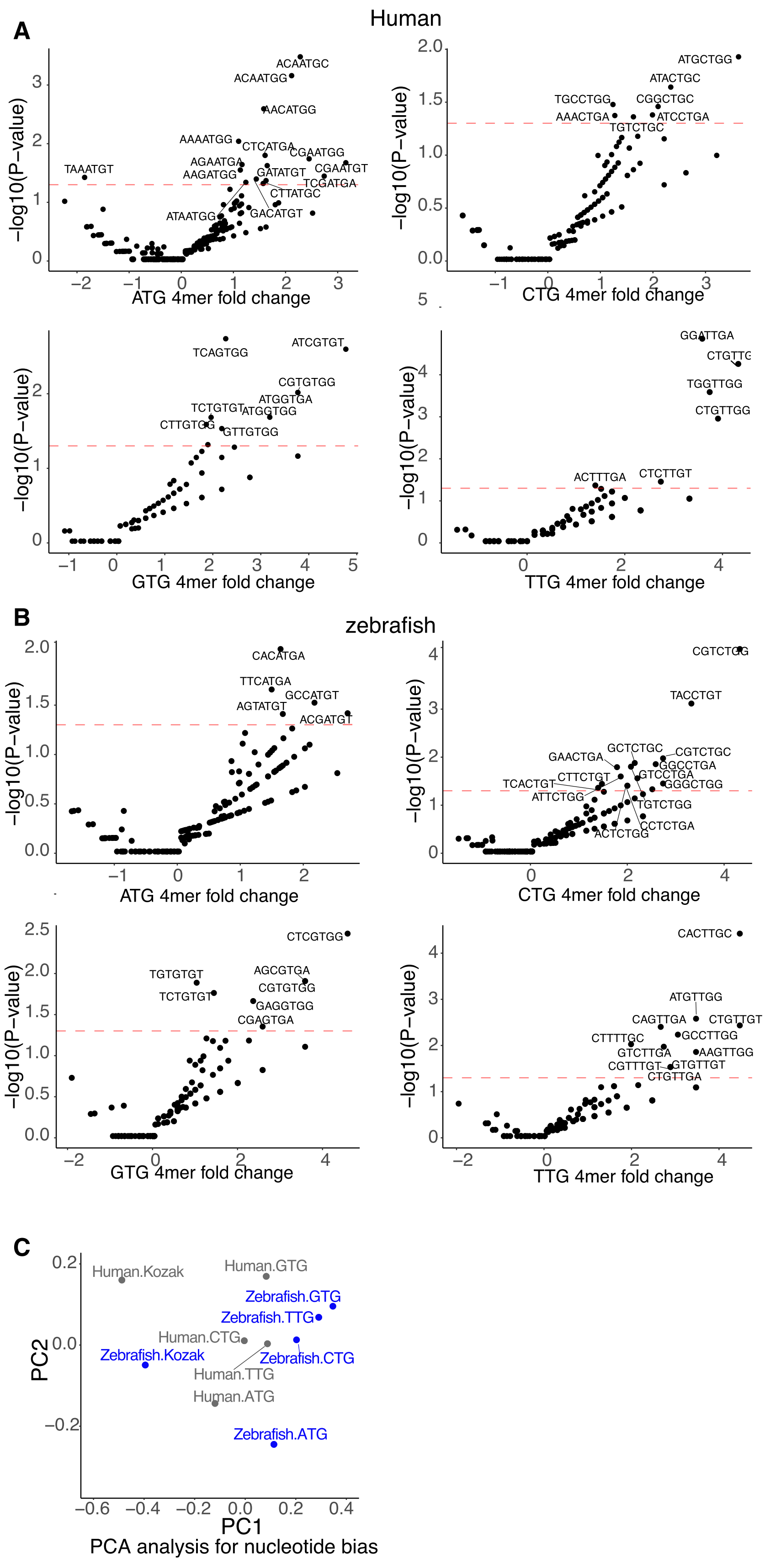

Figure EV5. Sequence bias nearby the dORF translation start codon.

A Volcano plot for the fold change and *P*-value of 4-mer (three nucleotides upstream and the first downstream of the dORF start codon) between translated and untranslated dORFs for human dORFs for each start codon, binomial test. Dots with annotated sequences are significantly biased in translated dORF; red dashed line is *P*-value 0.05.

B Volcano plot showing enrichment/depletion of the 4-mer (three nucleotides upstream and the first downstream of the dORF start codon) between translated and untranslated dORFs (log_2 fold change, *x*-axis), and *P*-value (*y*-axis), for zebrafish dORF for each of the translation start site codon (NTG), binomial test. Dots with annotated sequences are significantly biased in translated dORF; red dashed line is *P*-value 0.05.

C PCA for human and zebrafish dORF with different start codons based on the nucleotide bias of sequence nearby dORF start codon; the Kozak sequence indicates sequence nearby the canonical ORF translation start codon.

 © 2020 The Authors
