# Supplementary material for: Translation of small downstream ORFs enhances translation of canonical main open reading frames
Source: EMBO J. 2020 Aug 3;39(17):e104763. doi: 10.15252/embj.2020104763 (PMC7459409; doi:10.15252/embj.2020104763)
Supplement: Supplementary file 7 — Table EV1 [file EMBJ-39-e104763-s007.docx]

| **GO term** | **Description** | [P-value](http://cbl-gorilla.cs.technion.ac.il/GOrilla/zvf8bt29/GOResults.html#p_value_info) | [FDR q-value](http://cbl-gorilla.cs.technion.ac.il/GOrilla/zvf8bt29/GOResults.html#fdr_info) |
| --- | --- | --- | --- |
| [GO:0000981](http://www.godatabase.org/cgi-bin/amigo/go.cgi?query=GO:0000981&view=details) | DNA-binding transcription factor activity, RNA polymerase II-specific | 3.37E-07 | 7.60E-04 |
| [GO:0003700](http://www.godatabase.org/cgi-bin/amigo/go.cgi?query=GO:0003700&view=details) | DNA-binding transcription factor activity | 1.63E-06 | 1.84E-03 |
| [GO:1990837](http://www.godatabase.org/cgi-bin/amigo/go.cgi?query=GO:1990837&view=details) | sequence-specific double-stranded DNA binding | 1.47E-04 | 1.11E-01 |
| [GO:0140110](http://www.godatabase.org/cgi-bin/amigo/go.cgi?query=GO:0140110&view=details) | transcription regulator activity | 2.02E-04 | 1.14E-01 |
| [GO:0000976](http://www.godatabase.org/cgi-bin/amigo/go.cgi?query=GO:0000976&view=details) | transcription regulatory region sequence-specific DNA binding | 5.61E-04 | 2.53E-01 |
| [GO:0003690](http://www.godatabase.org/cgi-bin/amigo/go.cgi?query=GO:0003690&view=details) | double-stranded DNA binding | 5.83E-04 | 2.19E-01 |
| [GO:0003677](http://www.godatabase.org/cgi-bin/amigo/go.cgi?query=GO:0003677&view=details) | DNA binding | 9.65E-04 | 3.11E-01 |

Table EV1 GO analysis of human genes with translated dORFs
